# Cryo-EM structures of adenosine receptor A_3_AR bound to selective agonists

**DOI:** 10.1038/s41467-024-47207-6

**Published:** 2024-04-16

**Authors:** Hongmin Cai, Shimeng Guo, Youwei Xu, Jun Sun, Junrui Li, Zhikan Xia, Yi Jiang, Xin Xie, H. Eric Xu

**Affiliations:** 1 State Key Laboratory of Drug Research, Shanghai Institute of Materia Medica, Chinese Academy of Sciences, Shanghai, China; 2 University of Chinese Academy of Sciences, Beijing, China; 3 Lingang Laboratory, Shanghai, China; 4 School of Pharmaceutical Science and Technology, Hangzhou Institute for Advanced Study, University of Chinese Academy of Sciences, Hangzhou, China; 5 School of Life Science and Technology, ShanghaiTech University, Shanghai, China; 6 Shandong Laboratory of Yantai Drug Discovery, Bohai Rim Advanced Research, Institute for Drug Discovery, Yantai, China

**Keywords:** Single-molecule biophysics, Chemical biology, Biochemistry, Structural biology

## Abstract

The adenosine A_3_ receptor (A_3_AR), a key member of the G protein-coupled receptor family, is a promising therapeutic target for inflammatory and cancerous conditions. The selective A_3_AR agonists, CF101 and CF102, are clinically significant, yet their recognition mechanisms remained elusive. Here we report the cryogenic electron microscopy structures of the full-length human A_3_AR bound to CF101 and CF102 with heterotrimeric G_i_ protein in complex at 3.3-3.2 Å resolution. These agonists reside in the orthosteric pocket, forming conserved interactions via their adenine moieties, while their 3-iodobenzyl groups exhibit distinct orientations. Functional assays reveal the critical role of extracellular loop 3 in A_3_AR’s ligand selectivity and receptor activation. Key mutations, including His^3.37^, Ser^5.42^, and Ser^6.52^, in a unique sub-pocket of A_3_AR, significantly impact receptor activation. Comparative analysis with the inactive A_2A_AR structure highlights a conserved receptor activation mechanism. Our findings provide comprehensive insights into the molecular recognition and signaling of A_3_AR, paving the way for designing subtype-selective adenosine receptor ligands.

## Abstract (web summary)

Adenosine A_3_ receptor (A_3_AR) holds promise for treating inflammatory and cancer conditions. Here, Cai et al. present cryo-EM structures of  A_3_AR bound to agonists CF101 and CF102, offering insights into its activation and ligand interaction, crucial for developing targeted therapies.

## Introduction

The adenosine receptor subfamily of G protein-coupled receptors consists of four subtypes: A_1_, A_2A_, A_2B_, and A_3_^1,2^. These receptors are activated by the endogenous ligand, adenosine, to transduce downstream signals that mediate a number of important physiological and pathological functions including immunomodulation, energy balance, cardiac function, and neuroprotection^3–5^. The gene of A_3_AR was firstly cloned in 1991^6^ and characterized as a subtype of adenosine receptor in 1993^1^. It is expressed in various tissues including the brain, heart, lungs, liver, kidneys, and immune cells^7^. A_3_AR participates in regulating cardiac function, vasodilation, inhibition of inflammation, protection against ischemia-reperfusion injury, and suppression of oxidative stress. Additionally, A_3_AR is highly expressed in several tumor types, making it as a promising therapeutic target for suppressing cancer cell proliferation^7–9^.

A_1_AR and A_3_AR preferentially couple to the inhibitory G protein (G_i_), leading to the suppression of adenylate cyclase activity and a reduction in intracellular cyclic AMP levels, contrasting with the stimulatory G protein (G_s_) signaling triggered by A_2A_AR and A_2B_AR activation^2^. The structure of adenosine has inspired the design of various agonists and antagonists targeting A_3_AR, particularly for cancer, inflammation, and pain management^10^. Studies highlight that alterations at the N^6^ position of the purine ring and the 5’-N position of the ribose group enhance the potency and selectivity of A_3_AR agonists^11–13^. Notably, N^6^-methyladenosine (m^6^A), a methylated adenosine metabolite, emerged as a potent A_3_AR agonist^14^. CF101 and CF102 are representatives of such modification strategy with similar nucleoside core structure and only one chloro-substituent difference, both demonstrate high affinity and selectivity for A_3_AR^15–17^. These effective orally compounds have shown promise in disrupting key signaling pathways in cancer and inflammatory cells^10^. CF101 has demonstrated efficacy in Phase III trials for psoriasis and rheumatoid arthritis, while CF102 is being evaluated for hepatocellular carcinoma and non-alcoholic steatohepatitis^18,19^. The broad expression of adenosine receptors poses challenges in designing subtype-selective compounds^20,21^. The lack of structural information for A_3_AR, unlike other adenosine receptor subtypes, limits our understanding of its specific signaling mechanisms and impedes structure-based drug design.

Here, we present the cryo-EM structures of A_3_AR bound to the G_i_ protein in the presence of CF101 and CF102. These structures reveal the mechanisms of ligand recognition and activation in A_3_AR, providing valuable insights for designing effective, targeted therapies for conditions like cancer and inflammation.

## Results and discussion

### Overall structures of the complexes

**Fig. 1:**
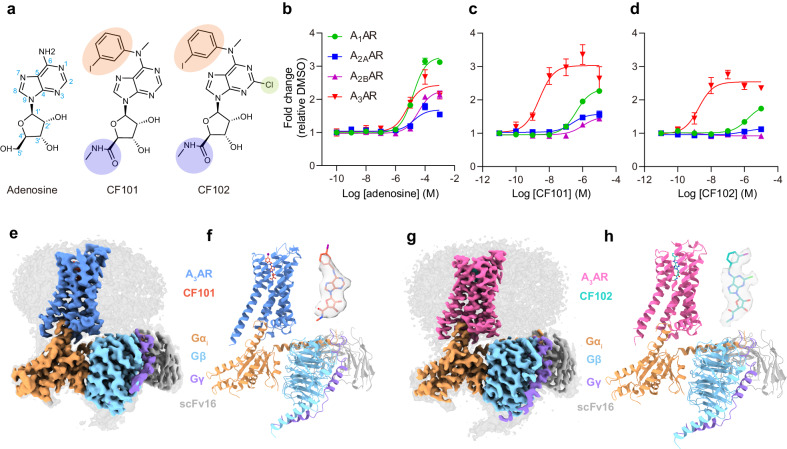
Cryo-EM structures of CF101-A_3_AR-G_i_ and CF102-A_3_AR-G_i_ complexes. **a** Chemical structures of the adenosine, CF101 and CF102 are provided, highlighting modifications at the 5’-N-methylcarboxamide in the ribose group, as well as the N^6^ and C2 positions of the adenosine group. The atom numbering is indicated in blue. CF101, is also named IB-MECA and N^6^-(3-iodobenzyl)adenosine-5’-N-methyluronamide. CF102, is also named Cl-IB-MECA and 2-chloro-N^6^-(3-iodobenzyl)adenosine-5’-N-methyluronamide. NanoBiT association assays monitoring ligand activity on adenosine receptors for adenosine (**b**), CF101 (**c**) and CF102 (**d**), respectively. Data shown are mean ± S.E.M. of three independent experiments (*n* = 3). Source data are provided as a Source Data file. Cryo-EM map (**e**) and model (**f**) of the CF101-A_3_AR-G_i_ complex, with inset showing CF101 density. The density map in the inset is shown at 0.232 threshold. Cryo-EM map (**g**) and model (**h**) of the CF102-A_3_AR-G_i_ complex, with inset showing CF102 density. The density map in the inset is shown at 0.17 threshold. Subunits are colored as indicated.

CF101 and CF102 are A_3_AR agonists that contain modifications to the ribose and adenine moieties, which confer their potent binding to A_3_AR. Specifically, CF101 and CF102 have a 5’-N-methylcarboxamide substitution on the ribose group and a N^6^-(3-iodobenzyl) substitution on the adenine base (Fig. 1a). These combined modifications result in significantly higher A_3_AR potency compared to the endogenous A_3_AR agonist adenosine. To ensure the specificity of our experiments in the context of HEK293 cells, which are known to express high levels of A_1_AR, A_2A_AR, and A_2B_AR but not A_3_AR, we employed NanoBiT association assays. These assays were crucial in determining the selectivity of CF101 and CF102 for A_3_AR, as opposed to other adenosine receptor subtypes (Fig. 1b–d). While adenosine activated four subtypes with similar micromolar potencies, CF101 and CF102 displayed strong potency of ~3 nM on A_3_AR but had weak or negligible response on other subtypes of adenosine receptors.

We used HiBiT tether approach to stabilize the full-length A_3_AR-G protein complexes, as it has been used for many GPCR structural studies^22–24^ (Supplementary Fig. S1). The large NanoLuc domain (LgBiT) and small high affinity fragment (HiBiT) was fused at the C-terminal of A_3_AR and Gβ, respectively. Meanwhile, A_3_AR used in this study had an N-terminal thermostabilized apocytochrome b562RIL (BRIL) fusion to enhance its expression, which is co-expressed with G protein subunits and scFv16, an antibody fragment that is used to further stabilize the receptor G protein complex. For the CF101-A_3_AR-G_i_ complex, data from 20,779 movies comprising 271,323 particles was used to determine the structure at 3.29 Å resolution (Supplementary Fig. S2, Supplementary Table S1). For the CF102-A_3_AR-G_i_ complex, data from 13,581 movies yielding 283,561 particles was used to determine the structure at a resolution of 3.19 Å (Supplementary Fig. S3, Supplementary Table S1). The structures of the CF101/CF102-A_3_AR-G_i_ complexes revealed that the ligands occupy the orthosteric binding pocket, with the core structures modeled clearly into the cryo-EM density at the center of the receptor transmembrane helical domain (TMD) (Fig. 1e–h).

The structures showed the canonical seven-transmembrane architecture for A_3_AR, with the intracellular domains occupied by the α5 helix of Gα_i_ for G_i_ coupling. The density maps enabled modeling of most of the structures, except for A_3_AR N-terminus residues M1-L8, third intracellular loop N211-Y222, C-terminus V301-E318, and the alpha-helical domain of Gα_i_. The extracellular loop M151-S165 was also less defined but the backbone could be established (Supplementary Fig. S4). Aside from these regions, the models were well-resolved. Overall, the two agonist-bound complexes were highly similar, with 0.593 Å root mean square deviation (RMSD) for the whole receptor.

### Binding mode of CF101/CF102 in A_3_AR orthosteric site

**Fig. 2:**
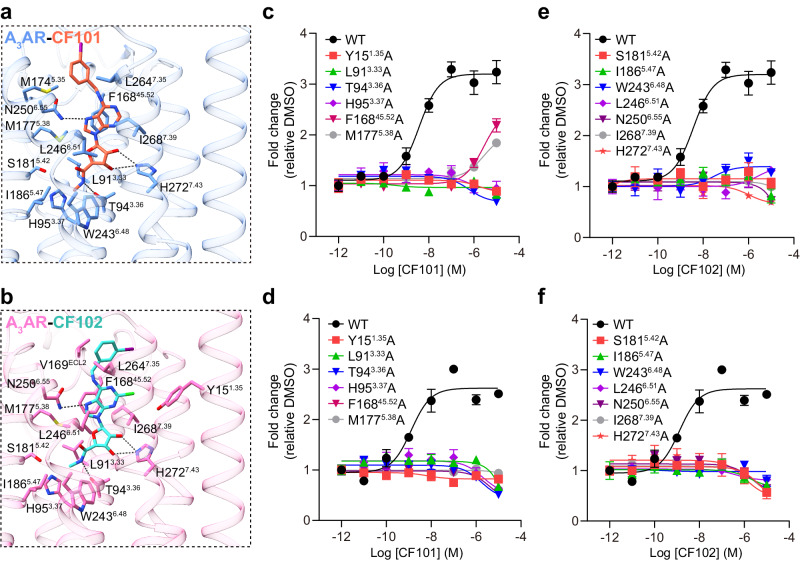
The orthosteric binding pocket. Detailed interactions between A_3_AR and CF101 (**a**) or CF102 (**b**) from the membrane plane. Residues involved in ligand interaction are colored blue and pink in two complexes, respectively. Black dashed lines indicate hydrogen bonds. Dose-response curves of mutants of A_3_AR induced by CF101 (upper panels, **c**, **e**) or CF102 (lower panels, **d**, **f**) using NanoBiT assay. Data shown are mean ± S.E.M. of three independent experiments (*n* = 3). Source data are provided as a Source Data file.

The A_3_AR agonists CF101 and CF102 bind at conserved orthosteric pocket forms by ECL2, TM3, TM5, TM6 and TM7, akin to the endogenous ligand adenosine bound to other adenosine receptor subtypes (Fig. 2a, b). However, the orientations of the modified 3-iodobenzyl moieties differ between CF101 and CF102. The adenine core mediates conserved receptor interactions commonly seen in other adenosine receptors^23,25,26^. Notably, the adenine pyrimidine forms π-stacks against F^45.52^, and the F^45.52^A mutation greatly affected the ability of CF101/CF102 to induce the receptor activation in the NanoBiT association assay (Fig. 2c–f, Supplementary Table S2). Additionally, 2’ and 3’ hydroxyl group in ribose and purine group form hydrogen bonds with polar side chains at positions 3.36, 6.55 and 7.43, which are conserved and critical for recognition of nucleoside ligands by adenosine receptors (Fig. 2c–f, Supplementary Table S2).

The ligand binding pocket is mainly comprised of hydrophobic residues, including position 3.33, 5.38, 5.47, 6.48, 6.51 and 7.39, which form hydrophobic contacts that are important for CF101/CF102 potencies (Fig. 2c–f, Supplementary Table S2). Alanine mutations at these positions severely reduced agonists’ ability to induce receptor activation. His^3.37^ and Ser^5.42^ participate van der Waals contacts with the bound ligands, their alanine mutations also affected activity (Fig. 2c–f, Supplementary Table S2). To confirm the functional data, cAMP accumulation assays were carried out to assess the agonist activity (Supplementary Fig. S5, Supplementary Table S3). The results from the NanoBiT association assay and cAMP accumulation assay were consistent. The side chains from M174^5.35^ and L264^7.35^ in the receptor form hydrophobic interactions with the 3-iodobenzyl group extended from the N^6^ position of the adenosine base of CF101. In contrast, the corresponding group of CF102 is surrounded by V169^ECL2^ and L264^7.35^ from the receptor. Alanine mutations on these residues did not significantly affect the potency of the compounds on A_3_AR (Supplementary Fig. S6, Supplementary Table S2), suggesting that the 3-iodobenzyl substituents may exist alternative states at the receptor extracellular domains. This demonstrates that the N^6^ position may accommodate various substituted groups through distinct conformations in the A_3_AR pocket.

CF102 is a 2-chloro derivative of CF101. In CF102-bound A_3_AR, Y15^1.35^ is situated near the 2-chloro group of CF102 (Supplementary Fig. S7a). The Y15^1.35^A mutation in A_3_AR abolished the agonist activity of both CF101 and CF102 (Fig. 2c, d). However, the Y15^1.35^F mutant only slightly impacted the potency of CF101 and CF102 (Supplementary Fig. S7b, c). Y15^1.35^ forms extensive π-π contact with Y265^7.36^ in TM7. The Y265^7.36^A mutant also affected the receptor’s ability to bind CF101 or CF102 (Supplementary Fig. S7). This implies that Y15^1.35^ likely plays a critical role in maintaining the stability and structural integrity of A_3_AR, thus affecting both CF101 and CF102 binding to the receptor. Additionally, modifications at the 2-position of adenosine tend to be well tolerated for A_3_AR binding^16^, whether incorporating a small or large substituent, or even linking it to the N^6^ moiety to form a macrocycle^27^. Elucidation of these subtle ligand and receptor interaction variations thus provides molecular insight into the conformational adaptability and binding poses governing molecular recognition by A_3_AR.

### The role of ECL3 in A_3_AR subtype selectivity

**Fig. 3:**
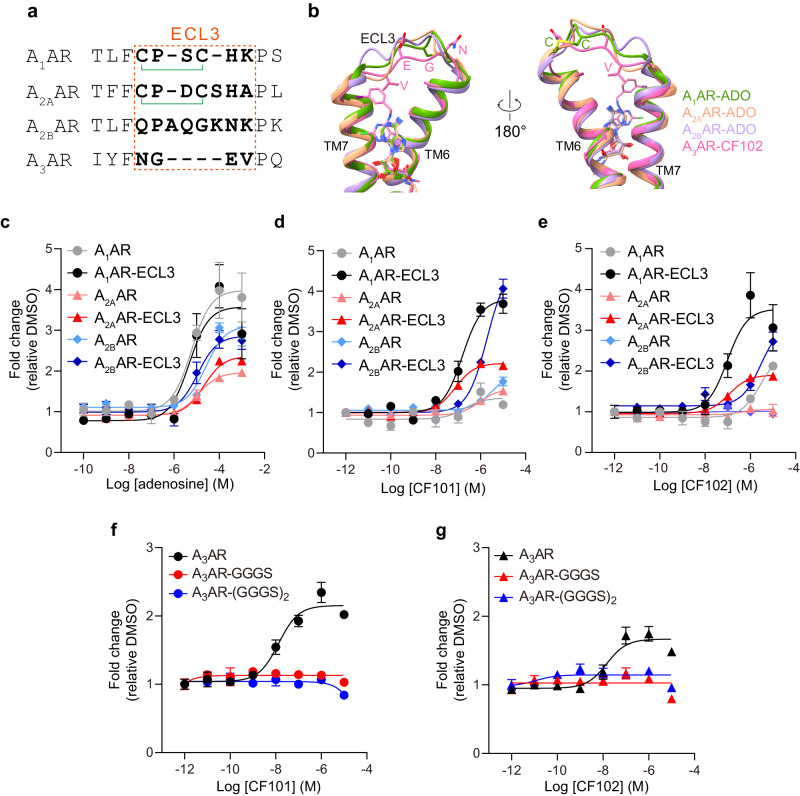
Swapping ECL3 on adenosine receptor subtypes. **a** sequence alignment of ECL3 among adenosine receptors. The disulfide bond was shown as green linker. **b** Superimposed structures of adenosine receptors reveal that A_3_AR has the shortest ECL3. The residues in A_3_AR are shown in pink. The residues formed disulfide bond on ECL3 in A_1_AR were shown in green. Other TMs were omitted. **c**–**e** Assessing the effects of adenosine, CF101, and CF102 on A_1_AR, A_2A_AR, and A_2B_AR, along with their corresponding mutants containing the swapped ECL3 from the A_3_AR using NanoBiT assays. The results were from three independent experiments. Data shown are mean ± S.E.M. of three independent experiments (*n* = 3). Source data are provided as a Source Data file. **f**, **g** Assessing the effects of CF101 and CF102 on A_3_AR and its mutants with flexible ECL3 using NanoBiT assays. Data shown are mean ± S.E.M. of three independent experiments (*n* = 3). Source data are provided as a Source Data file.

CF101 and CF102 show high selectivity on A_3_AR rather than other subtypes (Fig. 1c, d). Analysis the sequence of adenosine receptors reveals strong conservation within TMs, while the extracellular loops diverge among subtypes (Supplementary Fig. S8). ECL1 shows relatively distant from the orthosteric site. The residue F168^45.52^ in ECL2 of adenosine receptors provides critical π-π interactions with both agonists and antagonists binding to these receptors. However, A_3_AR possesses a shorter ECL3 than other subtypes (Fig. 3a). The shorter ECL3 may rigidify A_3_AR to minimize its conformational changes for ligand binding (Fig. 3b).

To assess the role of ECL3 in A_3_AR, we engineered chimeric receptors by grafting ECL3 from A_3_AR onto the backbones of other adenosine receptors. NanoBiT assays were performed to test the binding abilities of adenosine, CF101, and CF102 to wide-type or chimeric adenosine receptors (Fig. 3c–e). The result showed that the three chimeric adenosine receptors did not show increased binding ability to the endogenous ligand adenosine (Fig. 3c, Supplementary Table S4). However, the three ECL3-chimeric receptors gained the ability to bind CF101 and CF102 with increased efficacy or potency (Fig. 3d, e, Supplementary Table S3). These findings suggest that ECL3 could serve as a structural factor mediating the selective recognition CF101 and CF102 by A_3_AR. The significance of ECL3’s length and amino acid composition in A_3_AR’s ligand binding was further investigated through mutations. We mutated the original four ECL3 residues of A_3_AR to GGGS or (GGGS)_2_ that has the same length as ECL3 of A_2B_AR. Neither mutant above showed any binding ability to CF101 and CF102 (Fig. 3f, g), suggesting that both the specific length and the unique amino acid sequence of ECL3 play critical roles in the selective binding of ligands to A_3_AR, underscoring the complexity of ligand-receptor interactions in this context.

The proximity of the ECL3 to the N^6^ position in adenosine is likely a crucial factor in the selectivity of A_3_AR for N^6^-modified adenosine derivatives, as indicated by structure-activity relationship (SAR) studies^15,16^. Substituents at the N^6^ position, whether too small or overly bulky, can adversely affect the potency and affinity of ligands for A_3_AR. This relationship underscores the importance of ECL3 in ligand recognition, as the N^6^ position extends into A_3_AR’s binding pocket near ECL3. Understanding these intricate structural interactions is key for discerning the selectivity mechanisms of structurally similar ligands at different adenosine receptors.

### Residues in binding pocket across adenosine receptors

**Fig. 4:**
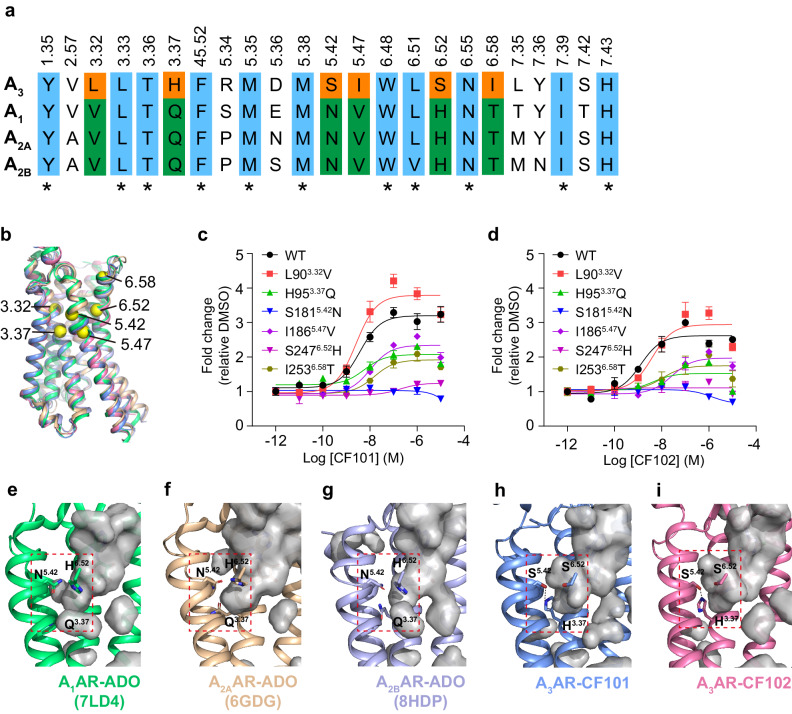
Key residues in the A_3_AR binding pocket. **a** Aligning the residues in the orthosteric binding pocket among the adenosine receptors. The conserved residues were colored in blue, and stars were used as markers. The unique residues in A_3_AR, distinct from other adenosine receptors subtypes were colored in orange, while residues in corresponding positions in other subtypes were colored in green. All residues were annotated based on GPCR Ballesteros-Weinstein numbering scheme. **b** In the superposition of adenosine receptors, the unique residues in A_3_AR, distinct from those in other adenosine receptors, are represented as yellow spheres. **c**, **d** Effects of CF101/CF102 on A_3_AR mutants containing swapped residues from other adenosine receptors by NanoBiT assay. Data shown are mean ± S.E.M. of three independent experiments (*n* = 3). Source data are provided as a Source Data file. **e**–**i** The binding cavities of the adenosine receptors were generated in PyMOL and depicted in gray. In A_3_AR, a subpocket is formed by His^3.37^, Ser^5.42^, and Ser^6.52^, while these positions are conserved as Gln^3.37^, Asn^5.42^, and His^6.52^ in other adenosine receptor subtypes (His, H; Ser, S; Gln, Q; Asn, N). In **h** and **i**, dashed lines depict the hydrogen bonds between His^3.37^ and Ser^5.42^. The names of the receptors and their associated PDB codes^23,26,45^ are indicated below each model.

Among adenosine receptors, A_3_AR stands out with the lowest sequence identity compared to other subtypes. This distinction is particularly evident in the orthosteric binding pocket (Fig. 4a), where A_3_AR’s unique residues at specific positions contribute to its selective ligand binding. Notably, positions 3.32, 3.37, 5.42, 5.47, 6.52, and 6.58 feature different amino acids in A_3_AR compared to A_1_, A_2A_, and A_2B_ receptors (Fig. 4b, Supplementary Fig. S9). Mutations at these positions to their counterparts in other subtypes were conducted to evaluate their impact on CF101 and CF102 binding and activity. NanoBiT assays and cAMP accumulation assays were utilized to cross-confirm the effects of the mutations (Fig. 4c, d, Supplementary Fig. S10, Supplementary Tables S2 and 3).

We found that changing the leucine at 3.32 to valine, similar to other subtypes, had no significant effect on the activity of CF101 and CF102 in NanoBiT assay, likely due to their comparable hydrophobic properties (Fig. 4c, d). However, mutations at positions 5.47 and 6.58 altered the receptor activation, indicating the importance of side chain length at these positions for ligand binding (Fig. 4c, d).

Furthermore, the hydrogen bond formation between H95^3.37^ and S181^5.42^ in A_3_AR, which was absent in other subtypes, appears critical (Fig. 4e–i). Mutations H95^3.37^Q and S247^5.42^N significantly impacted CF101 and CF102 activities (Fig. 4c, d), highlighting the importance of these residues in ligand-receptor interaction. The mutation of S247^6.52^ to histidine also reduced ligand activity, suggesting the influence of steric and electronic properties of the side chains (Fig. 4c, d, Supplementary Table S2).

Residues H95^3.37^, S181^5.42^ and S247^6.52^ form a unique sub-pocket in A_3_AR to accommodate the 5’-N-methylcarboxamide from the ribose (Fig. 4h, i, Supplementary Fig. S11). The mutational results implicate this sub-pocket might serve as a structural determinant for stabilizing CF101 and CF102 in A_3_AR versus other subtypes. Our results above with NanoBiT assay were replicated with traditional cAMP accumulation assays (Fig. 4c, d, Supplementary Fig. S10), further demonstrating that how minor sequence variations in receptors can significantly influence their conformations and ligand binding specificity.

### Activation mechanisms of A_3_AR

**Fig. 5:**
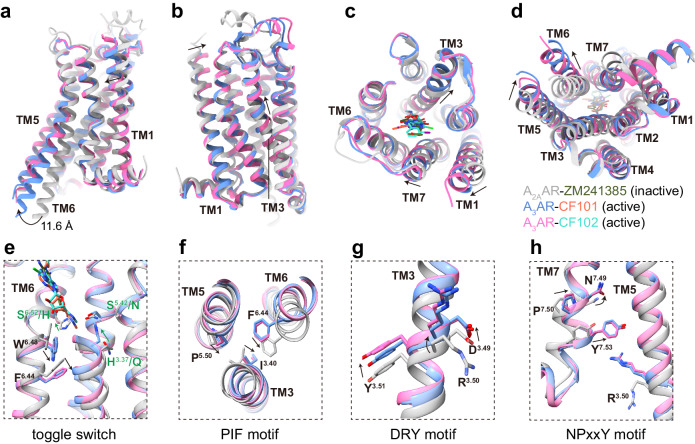
A_3_AR activation mechanism. **a**, **b** Superposition of active A_3_AR-CF101/CF102 complexes (blue/pink) with inactive A_2A_AR-ZM241385 complex (gray, PDB ID 4EIY). Comparison of extracellular (**c**) and cytoplasmic (**d**) views of active A_3_AR and inactive A_2A_AR. **e**–**h** Conformational changes in conserved motifs, including the toggle switch, PIF, DRY and NPxxY, upon CF101/CF102 binding to A_3_AR relative to inactive state of A_2A_AR-ZM241385. Arrows indicate movement directions. In **e**, The sub-pocket in A_3_AR is formed by residues at position 3.37, 6.52 and 6.52. The residues at these positions from both A_3_AR and A_2A_AR were labeled in green.

Structural comparisons between active, agonist-bound A_3_AR complexes and an inactive, antagonist-bound A_2A_AR structure (PDB ID: 4EIY)^28^ reveal classical hallmarks of conformational changes associated with GPCR activation^29,30^. Notably, the A_3_AR structures exhibit an outward movement of TM6 compared to inactive A_2A_AR, shifting 11.6 Å based on measurements of residue Glu^6.30^ at Cα atoms in receptors (Fig. 5a). Additional rearrangements of activation include inward movements of TM1 and TM7 and an upward shift of TM3 in A_3_AR relative to inactive A_2A_AR (Fig. 5b–d).

Detail structural analysis also provide potential mechanism of ligand induced A_3_AR activation.

A unique sub-pocket formed by H^3.37^, S^5.42^ and S^6.52^ residues confers selectivity over other adenosine receptor subtypes (Fig. 5e). This facilitates deeper binding of CF101/CF102 compared to A_2A_AR antagonists, enabling engagement with conserved motifs like the W^6.48^ “toggle switch”. Propagation through P^5.50^I^3.40^F^6.44^, D^3.49^R^3.50^Y^3.51^, and N^7.49^P^7.50^xxY^7.53^ motifs transduces rearrangements (Fig. 5f–h), while limited ECL3 flexibility likely assists selective activation. By elucidating the structural transitions from inactive to active A_3_AR, our findings provide molecular insights connecting specialized agonist recognition to downstream signaling activation.

### G protein coupling of adenosine receptors

**Fig. 6:**
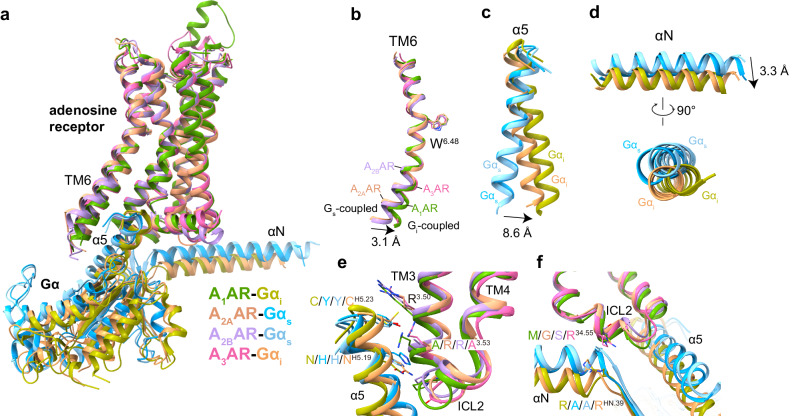
G protein coupling of adenosine receptors. **a** Comparison of adenosine receptor and Gα protein conformations in A_1_/A_3_AR-G_i_ and A_2A_/A_2B_AR-G_s_ complexes. Omitting the Gβ and Gγ subunits. The PDB codes for A_1_AR, A_2A_AR and A_2B_AR are 7LD4, 6GDG and 8HDP, respectively. **b** Conformational comparison of TM6 in adenosine receptors, with reference to the toggle switch residue W^6.48^ in TM6 of receptor. **c**, **d** Conformational comparison of the α5 helix and αN helix in G protein among adenosine receptor-G protein complexes. Arrows indicate movement directions. **e**, **f** Distinguishing residues on TM3 and ICL2 in adenosine receptor subtypes that participate in G protein coupling are highlighted. Components of adenosine receptors-G protein complexes are colored as indicated.

Adenosine receptors exhibit differential G protein coupling preferences that correlate with distinct conformational orientations of the associated G proteins^23,25,26^. Structural alignment reveals A_3_AR-G_i_ shares better overlay with A_1_AR-G_i_ versus A_2A_/A_2B_AR-G_s_ (Fig. 6a). The analogous G_i_-binding modes of A_3_AR and A_1_AR contrast A_2A_/A_2B_AR’s G_s_-coupling preferences, consistent with sequence and functional profiles. Notably, TM6 positioning facilitates differential G protein accommodation, 3.1 Å inward shift enables A_1_/A_3_AR-G_i_ versus A_2A_/A_2B_AR-G_s_ binding (Fig. 6b). Additionally, α5 helix and αN of G_i_ protein tilt orient differently between complexes, induced by receptors’ hydrophobic and polar residue interactions (Fig. 6c, d). The α5 helix of Gα_s_ subunits in A_2A_AR-G_s_ displays an 8.6 Å displacement relative to its orientation in A_3_AR-G_i_ complexes based on measurements of the Cα atom of Gα^H5.03^ (Fig. 6c). The αN helix of Gα_i_ exhibits a 3.3 Å tilt compared to G_s_ when measuring the Cα of Gα^HN.39^.

Furthermore, different adenosine receptors induced a variation in the N-terminal helix (αN) tilt of the Gα protein (Fig. 6d). The residue at position 34.51 (L/L/L/V, the residue in A_1_/A_2A_/A_2B_/A_3_-AR) in receptors is conserved as a hydrophobic residue that forms hydrophobic interactions with the G protein by inserting into the cleft between αN and α5 of the Gα protein (Supplementary Fig. S12a). Besides, sequence alignment of adenosine receptors showed that residues at positions 3.53 (R/A/A/R) and 34.55 (M/G/S/R) revealed different preferences in different subtypes (Fig. 6e, f, Supplementary Fig. S12a). The longer side chains in G_i_-coupled A_1_AR and A_3_AR likely triggered more noticeable translocations in the αN and α5 helix to accommodate the Gα_i_ protein. The main chains from R^3.53^ and P^34.50^ in A_1_AR formed a polar interaction with N348 in Gα_i_ protein (Supplementary Fig. S12b). The side chain of R^3.53^ and H^4.39^ in A_3_AR formed a polar interaction with the side chain of N347 and E28 in Gα_i_ protein, respectively (Supplementary Fig. S12c). Both of the complexes of A_2A_AR/A_2B_AR-G_s_, H41 in Gα_s_ formed a polar interaction with the main chain from the receptor’s ICL2 (Supplementary Fig. S12d, e). Together, these findings reveal that preferred G_i_-coupled adenosine receptors adopt conserved G_i_ protein-binding conformations that differ distinctly from those of G_s_-coupled adenosine receptor subtypes.

In summary, we have determined cryo-EM structures of the A_3_AR bound to selective agonists CF101 and CF102 with heterotrimeric G_i_ protein. Despite the conserved binding of the core adenosine moiety, the structures revealed differences in the orientations of the N^6^ substituted groups in CF101 and CF102. We have identified ECL3 and key sub-pocket residues His^3.37^, Ser^5.42^ and Ser^6.52^ that confer selectivity over other adenosine receptor subtypes by structural and mutational studies. Comparison to an inactive A_2A_AR structure provided insight into the conformational changes associated with A_3_AR activation and G protein coupling. By elucidating the molecular mechanisms governing ligand recognition, signaling, and subtype selectivity, the experimentally determined A_3_AR structures significantly advance our fundamental understanding of this important drug target. The findings pave the way for structure-guided design of selective ligands targeting adenosine receptors subtypes for the treatment of cancer, inflammation, and other diseases.

## Methods

### Construct design

The full-length gene coding human A_3_AR was synthesized (Synbio) and subcloned into pFastBac vector using ClonExpress II one step cloning kit (Vazyme Biotech, C112). A hemagglutinin signal peptide and thermostabilized apocytochrome b562RIL (BRIL) were fused at the N-terminal of A_3_AR to enhance receptor expression. To enhance complex stability, a NanoBiT tethering approach was used where an LgBiT domain was fused to the C-terminal of the receptor^22^. A dual maltose-binding protein was linked after LgBiT through a tobacco etch virus protease site (TEV site) for further cleavage. A dominant-negative mutant of bovine Gα_i_ containing G203A/A326S^31^ mutations was generated to stabilize the heterotrimeric Gα_i_βγ protein. Rat Gβ1 was fused with a HiBiT at C-terminal for structural complementation of LgBiT to form a NanoBiT. The single-chain variable fragement scFv16 was applied to bind the Gα_i_βγ protein for stabilization^32^. Gα_i_, Gβ1-HiBiT, Gγ, and scFv16, were cloned into pFastBac vector (Supplementary Fig. 1a), respectively.

### Protein expression and purification

The recombinant A_3_AR, Gα_i_, Gβ1-HiBiT, Gγ, and scFv16 were co-expressed in *Trichoplusia ni* High Five insect cells using the Bac-to-Bac baculovirus expression system. High Five cells were co-infected with the baculovirus at a cell density of 3.5 × 10^6^ cells per milliliter. Fourty-eight hours later, the infected cells were harvested and stored at −80 °C until used.

For the purification of the CF101-A_3_AR-G_i_ complex, cells pellets were thawed and resuspended in Buffer A (100 mM NaCl, 20 mM HEPES, pH 7.5) supplemented with protease inhibitor cocktail (TargetMol, C0001). Cells were lysed by dounce homogenization (Sigma-Aldrich, D9188) followed by centrifugation to remove unsoluble materials. The pellets were resuspended in Buffer B (100 mM NaCl, 10 %(v/v) glycerol, 20 mM HEPES, pH 7.5) supplemented with 10 mM MgCl_2_, 5 mM CaCl_2_, 0.2 mM Tris-(2-carboxyethyl)phosphine (TCEP, Hampton Research, HR2-801) and protease inhibitor cocktail. We formed the complexes by rotating the samples at room temperature for 1 h after addition of 25 mUnit/mL apyrase and 10 μM CF101 (MedChemExpress, HY-13591). After incubation, the sample was solublized in 0.5 %(w/v) lauryl maltose neopentylglycol (LMNG, anatrace, NG310) and 0.1%(w/v) cholesteryl hemisucinate (CHS, anatrace, CH210) for 3 h at 4 °C. The supernatant was clarified by centrifugation at 100,000× g for 40 min. The supernatant was incubated with dextrin beads 6FF (Smart-Lifesciences, SA02601L) for 3 h at 4 °C. The beads were loaded onto a gravity column and washed with 20 column volumes of Buffer C (100 mM NaCl, 2 mM MgCl_2_, 10 μM CF101, 0.2 mM TCEP, 0.01 %(w/v) LMNG, 0.002 %(w/v) CHS, 20 mM HEPES, pH 7.5). The complex was eluted with Buffer C supplemented with 10 mM maltose and further concentrated using 100 kDa molecular weight cut-off concentrator. TEV protease was added to the concentrated protein at 4 °C overnight to cleave dual maltose binding protein from fusion protein. After digestion, sample was loaded onto Superdex 200 Increase 10/300 GL column (Cytiva, 28-9909-44) with Buffer D (100 mM NaCl, 2 mM MgCl_2_, 10 μM CF101, 0.1 mM TCEP, 0.00075 %(w/v) LMNG, 0.00025 %(w/v) glyco-diosgenin, 0.0002 %(w/v) CHS, 20 mM HEPES, pH 7.5). The desired fractions were pooled and concentrated to 5–8 mg/mL for cryo-EM sample preparation. The purification procedures of CF102-A_3_AR-G_i_ complex were almost the same as in CF102-A_3_AR-G_i_ complex preparation, while the CF101 compounds was replaced by CF102 (TargetMol, T6884).

### Cryo-EM data collection

Cryo-EM grids were prepared with the Vitrobot Mark IV plunger (FEI) set to 8 °C and 100% humidity. Three-microliters of the CF101-A_3_AR-G_i_ complex were applied to glow- discharged Quantifoil R1.2/1.3 holey carbon grids. The sample was incubated for 10 s on the grids before blotting for 3.5 s (double-sided, blot force 1) and flash-frozen in liquid ethane immediately. The same conditions were used for the CF102-A_3_AR-G_i_ complex sample.

For CF101-A_3_AR-G_i_ complex, three datasets comprising 20,779 movies were collected on a Titan Krios equipped with a Gatan K3 direct electron detection device at 300 kV with a magnification of 105,000 corresponding to a pixel size 0.824 Å. Image acquisition was performed with EPU Software (FEI Eindhoven, Netherlands). We collected a total of 36 frames accumulating to a total dose of 50 e^−^ Å^−2^ over 2.5-s exposure.

For CF102-A_3_AR-G_i_ complex dataset, two datasets totaling 13,581 movies were collected on a Titan Krios equipped with a Gatan K3 detector at 300 kV with a magnification of 105,000 and pixel size of 0.824 Å, using EPU Software (FEI Eindhoven, Netherlands). Thirty-six frames were collected over a 2.5-s exposure to a dose of 50 e^−^ Å^−2^.

### Image processing

MotionCor2 was used to perform the frame-based motion-correction algorithm to generate drift-corrected micrograph for further processing, and CTFFIND4 provided estimation of contrast transfer function (CTF) parameters^33,34^.

For the CF101-A_3_AR-G_i_ dataset, the previously resolved structure of BAY 60-6583-A_2B_AR-G_s_^23^ was used as a reference for automatic particle picking in RELION 3.0^35^. Particle picking and extraction yielded 4,550,294 particles after 2D classification clearance, which were imported into CryoSPARC^36^. Four rounds of 2D classification selected 1,267,837 particles, followed by two rounds of 3D heterogenous refinement giving 982,833 particles. After two additional rounds of 2D classification and two rounds of heterogenous refinement, 271,323 particles were refined to a structure at 3.29 Å global resolution using non-uniform refinement (Supplementary Fig. 2).

For CF102-A_3_AR-G_i_ complex dataset, the BAY 60-6583-A_2B_AR-G_s_ structure^23^ was again used for reference-based particle picking. 4,090,959 and 4,833,382 particles were autopicked and extracted from Dataset 1 and Dateset 2, respectively. For Dataset 1, two rounds of 2D classification were used to separate out 1,070,085 particles. Masked 3D classification on the receptor part was used to separate out 175,747 particles that resulted to a clearer density of A_3_AR. For Dataset 2, two rounds of 2D classification and two rounds of 3D classification were performed to separate out 246,392 particles. After clearance, the remained particles from two datasets were combined and subjected to alignment-free 3D classification. 283,561 particles were remained and transferred in CryoSPARC^36^. One round of heterogenous refinement yielded a final 102,581 particles were refined to a structure at 3.19 Å global resolution using non-uniform refinement (Supplementary Fig. 3).

### Model building

An A_3_AR structure predicted by AlphaFold2 was used as the starting reference models for receptors building^37^. Structures of Gα_i_, Gβ, Gγ and the scFv16 were derived from PDB entry 7EZH^38^ were rigid body fit into the density. All models were fitted into the EM density map using UCSF Chimera^39^ followed by iterative rounds of manual adjustment and automated rebuilding in COOT^40^ and PHENIX^41^, respectively. The model was finalized by rebuilding in ISOLDE^42^ followed by refinement in PHENIX with torsion-angle restraints to the input model. The final model statistics were validated using Comprehensive validation (cryo-EM) in PHENIX and provided in the supplementary information, Supplementary Table 1. All structural figures were prepared using Chimera^39^, Chimera X^43^, and PyMOL (Schrödinger, LLC.).

### NanoBiT assay

To monitor G protein interaction with A_1_AR, A_2A_AR, A_2B_AR or A_3_AR upon agonist stimulation, a NanoLuc-based NanoBiT enzyme complementation assay was used as previously described^44^. The C terminus of A_1_AR, A_2A_AR or A_2B_AR was fused to SmBiT, while LgBiT was fused to the N terminus of miniG proteins. The C terminus of A_3_AR was fused with LgBiT, and the SmBiT was fused to the N terminus of miniG proteins. HEK293 cells were seeded at 4 × 10^4^ cells/well on 96-well plates and co-transfected with adenosine receptor-SmBiT and LgBiT-G protein plasmid. After 24 h, cells were replaced with 40 μL fresh culture medium without fetal bovine serum. Ten microliter Nano-Glo Live Cell reagent was added followed the manufacturer’s protocol (Promega, N2011), and incubated at 37 °C, 5 % CO_2_ for 5 min. Another 25 μL culture medium containing various concentrations of compounds were added and incubated at room temperature for 10 min before measuring bioluminescence using EnVision multiplate reader (PerkinElmer).

### cAMP accumulation assays

HEK293 cells expressing wide-type (WT) or mutant A_3_AR were harvested and resuspended in DMEM containing 500 μM 3-isobutyl-1-methylxanthine (IBMX) at a density of 2 × 10^5^ cells/ mL. Cells were then plated onto 384-well assay plates at 1000 cells/ 5 μL/ well. Another 5 μL buffer containing 1 μM Forskolin and various concentrations of test compounds were added to the cells. After incubation at room temperature for 15 min, intracellular cAMP level was tested by a LANCE Ultra cAMP kit (PerkinElmer, TRF0264) and EnVision multiplate reader according to the manufacturer’s instructions.

### Cell-surface expression assay

The same constructs were used in the cell-surface expression assays, NanoBiT assays, and cAMP measurements. A human influenza hemagglutinin tag (HA-tag) was fused to the N-terminus of the adenosine receptor and mutant gene sequences in the pcDNA3.0 vector constructs used across the various assays. HEK293 cells were transfected with wild type (WT) or adenosine receptor mutants and then were seeded at 4 × 10^4^ cells/well on 96-well plates. After 24 h, cells were washed with PBS buffer, fixed with 4 %(w/v) paraformaldehyde for 15 min, and blocked with 2 %(w/v) bovine serum albumin (BSA) for 1 h. Next, cells were incubated with the polyclonal anti-HA antibody (diluted at a ratio of 1:1,000, Sigma-Aldrich, H6908) overnight at 4 °C, followed by 1 h with horseradish peroxidase (HRP)-conjugated anti-rabbit antibody (diluted at a ratio of 1:10,000, Cell Signaling, 7074S) at room temperature. After washing, 50 μL tetramethylbenzidine (Sigma, T0440) was added for 30 min before stopping the reaction with 25 μL 3,3,5,5 - tetramethylbenzidine (TMB) substrate stop solution (Beyotime, P0215). Absorbance at 450 nm was measured on a FlexStation III microplate reader (Molecular Devices).

### Statistical analysis

All functional study data were analyzed in Prism 8 (GraphPad) and presented as means ± S.E.M. from at least three independent experiments. Concentration-response curves were evaluated with a three-parameter logistic equation. p*EC*_50_ values were calculated using the sigmoid three-parameter equation. Significance was determined by one-way ANOVA followed by multiple comparisons test, and *P < 0.05 *vs*. wild-type (WT) was considered statistically significant.

### Reporting summary

Further information on research design is available in the Nature Portfolio Reporting Summary linked to this article.

### Supplementary information

Supplementary Information

Peer Review File

Reporting Summary

### Source data

Source Data

## Data Availability

The data that support this study are available from the corresponding authors upon request. The cryo-EM maps have been deposited in the Electron Microscopy Data Bank (EMDB) under accession codes EMD-37985 (A_3_AR-CF101-G_i_ complex) and EMD-37986 (A_3_AR-CF102-G_i_ complex). The atomic coordinates have been deposited in the Protein Data Bank (PDB) under accession codes 8X16 [10.2210/pdb8X16/pdb] (A_3_AR-CF101-G_i_ complex) and 8X17 [10.2210/pdb8X17/pdb] (A_3_AR-CF102-G_i_ complex). Previously published structures can be accessed via accession codes 7LD4, 6GDG, 8HDP and 4EIY. Source data are provided with this paper.
