# Supplementary material for: Cryo-EM structures of adenosine receptor A3AR bound to selective agonists
Source: Nat Commun. 2024 Apr 16;15:3252. doi: 10.1038/s41467-024-47207-6 (PMC11021478; doi:10.1038/s41467-024-47207-6)
Supplement: Supplementary file 1 — Supplementary Information [file 41467_2024_47207_MOESM1_ESM.pdf]

Supplementary information

Cryo-EM structures of adenosine receptor A_3AR bound to selective agonists
Hongmin Cai[1,7]*, Shimeng Guo[1,7], Youwei Xu[1,7], Jun Sun[1,2,7], Junrui Li[1], Zhikan Xia[1], Yi Jiang[3], Xin Xie[1,2,4,5,6]*, H. Eric Xu[1,2,5]*

[1] State Key Laboratory of Drug Research, Shanghai Institute of Materia Medica, Chinese Academy of Sciences, Shanghai, China.
[2] University of Chinese Academy of Sciences, Beijing, China.
[3] Lingang Laboratory, Shanghai, China.
[4] School of Pharmaceutical Science and Technology, Hangzhou Institute for Advanced Study, University of Chinese Academy of Sciences, Hangzhou, China.
[5] School of Life Science and Technology, ShanghaiTech University, Shanghai, China.
[6] Shandong Laboratory of Yantai Drug Discovery, Bohai Rim Advanced Research Institute for Drug Discovery, Yantai, China
[7] These authors contributed equally: Hongmin Cai, Shimeng Guo, Youwei Xu, Jun Sun

***Correspondence:** caihongmin@simm.ac.cn (H.C.); xxie@simm.ac.cn (X.X.); eric.xu@simm.ac.cn (H.E.X.)

This file includes:
Fig.S1 to Fig.S12
Table S1 to S3
Supplementary References

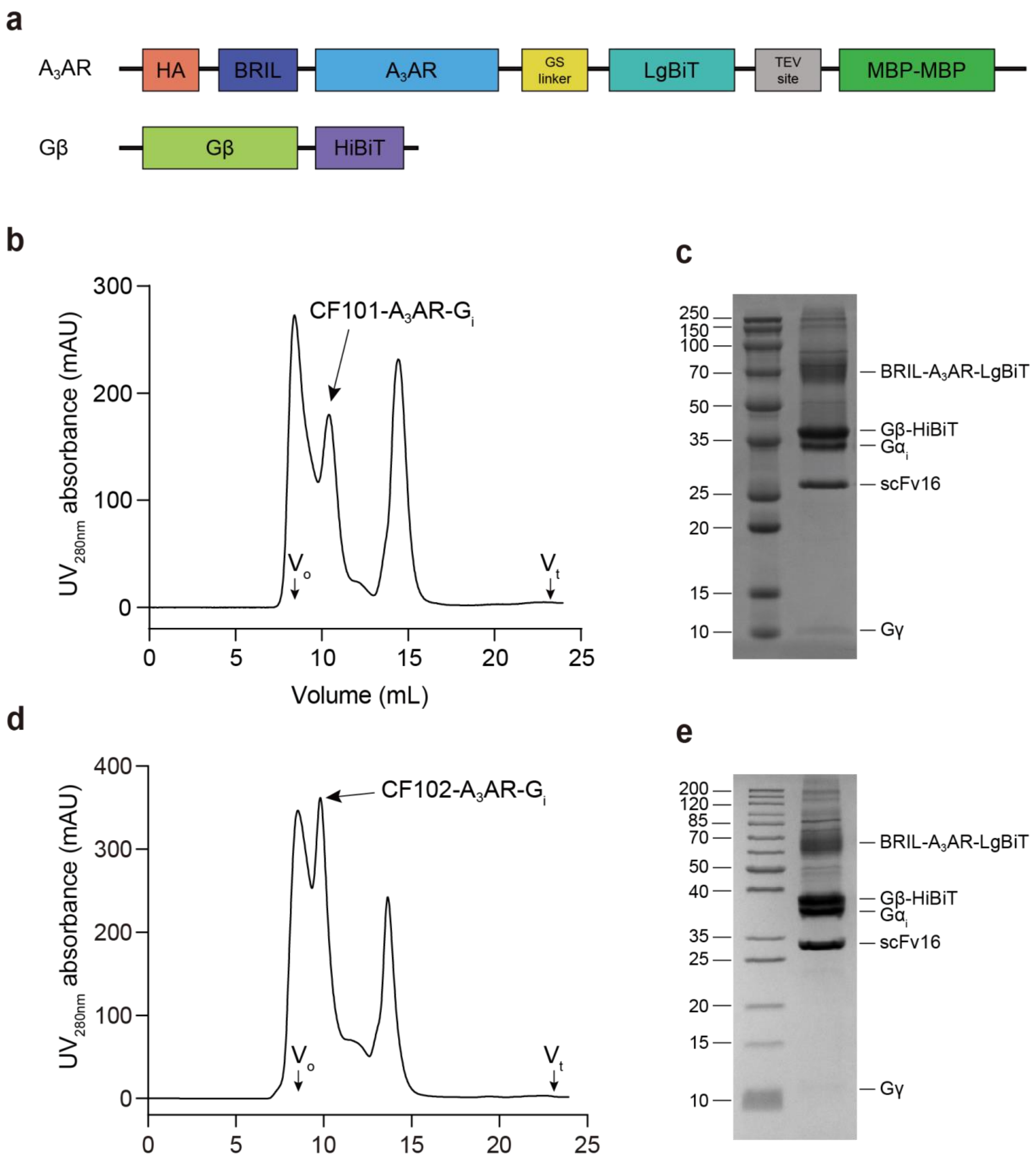

Fig. S1 The expression and purification of A_3AR-G_i complex.

a. Schematic diagrams of the expression constructs of A_3AR and Gβ1 using the NanoBiT tethering approach. A_3AR and Gβ1 fused with LgBiT and HiBiT, respectively. **b.** Size-exclusion chromatography profile of the CF101-A_3AR-G_i complex. **c.** SDS-PAGE of the arrow indicated peak fraction in (**b**). **d.** Size-exclusion chromatography profile of the CF102-A_3AR-G_i complex. **e.** SDS-PAGE of the arrow indicated peak fraction in (**d**).

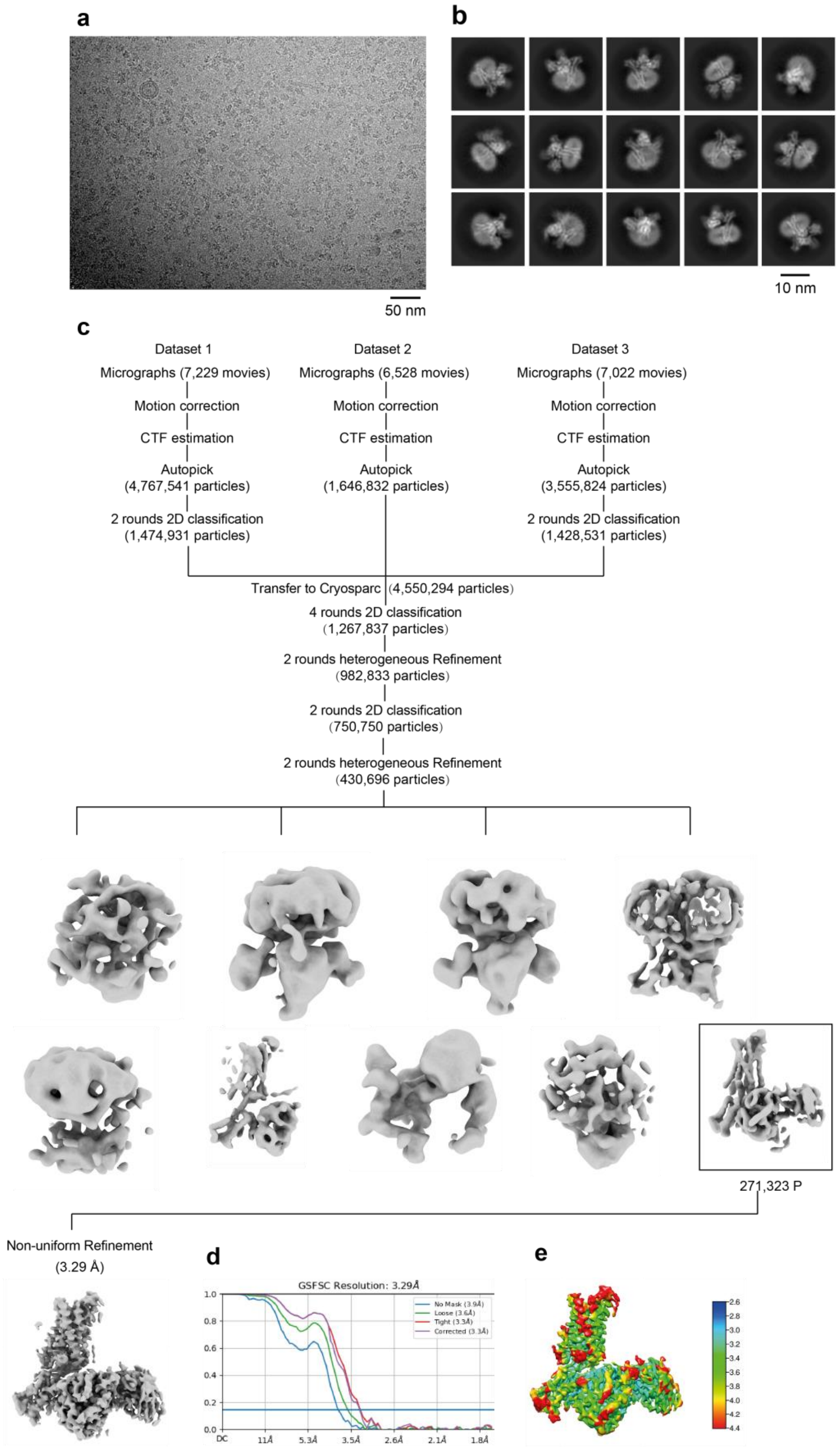

Fig. S2 Cryo-EM data processing of CF101-A$_3$AR-G$_i$ complex.

a. Representative image from cryo-EM dataset. Scale bar, 50 nm. **b.** Respresentative 2D average classification classes. Scale bar, 10 nm. **c.** Flow-chart of the cryo-EM data processing. **d.** FSC curves. **e.** The local resolution map.

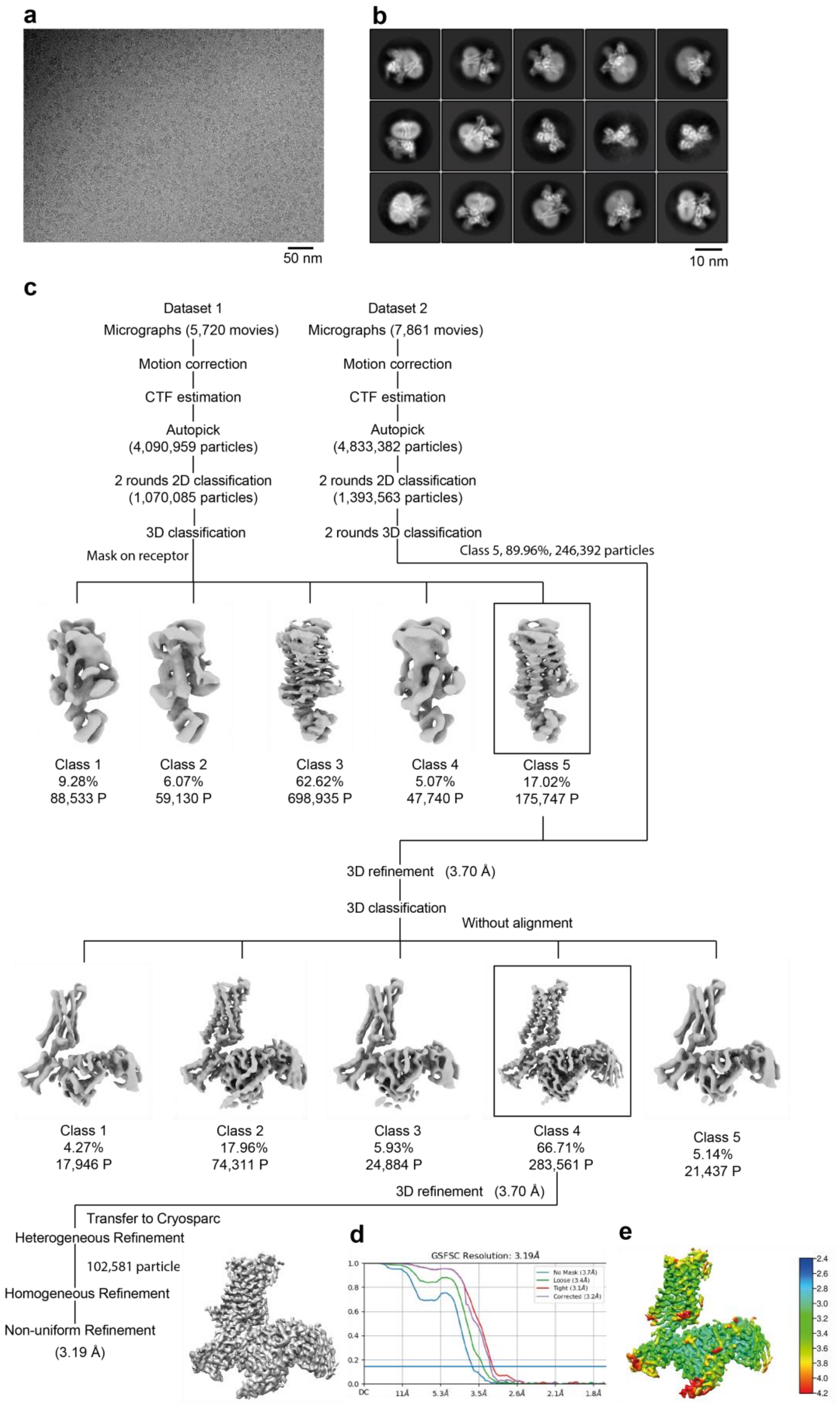

Fig. S3 Cryo-EM data processing of CF102-A$_3$AR-G$_i$ complex.

a. Representative image from cryo-EM dataset. Scale bar, 50 nm. **b.** Respresentative 2D average classification classes. Scale bar, 10 nm. **c.** Flow-chart of the cryo-EM data processing. **d.** FSC curves. **e.** The local resolution map.

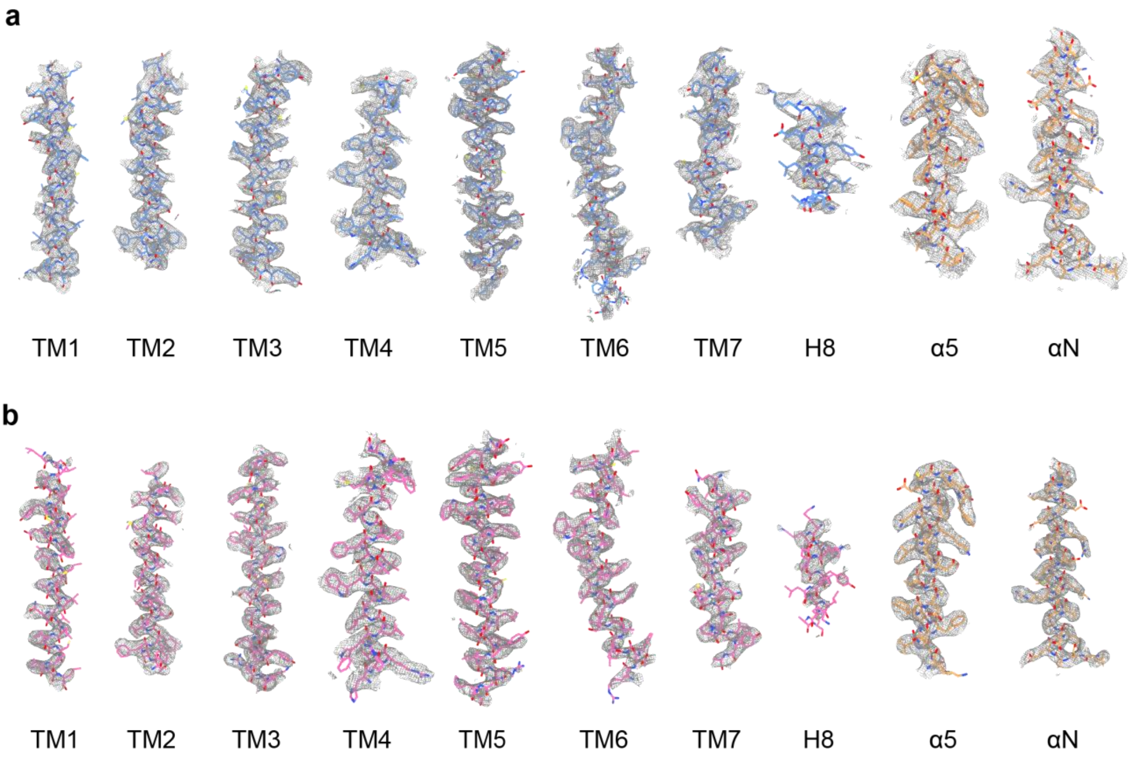

Fig. S4 Respresentative regions of cryo-EM density maps are shown for the each transmembrane helical (TM) of A_3AR and the α5 and αN helices of $G\alpha_i$.

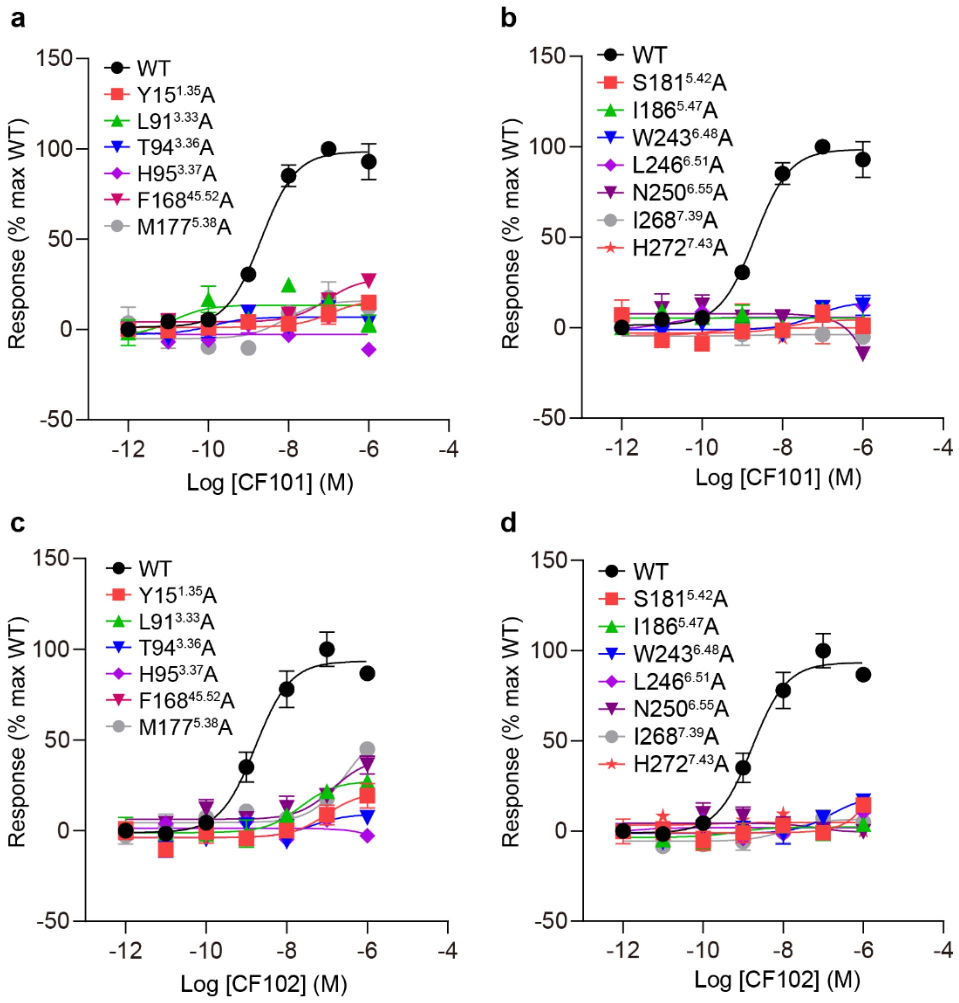

Fig. S5 CF101- and CF102-binding pocket of A_3AR. Dose-response curves of mutants of CF101 (**a** and **b**) and CF102 (**c** and **d**) in activating A_3AR with mutations in the binding pocket with cAMP accumulation assay. Data shown are mean ± S.E.M. of three independent experiments (n = 3). Source data are provided as a Source Data file.

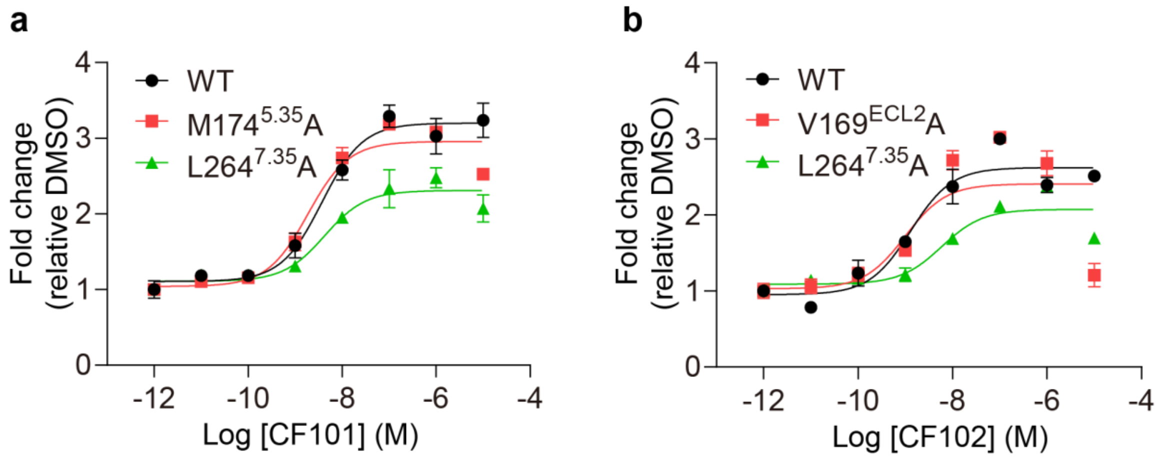

Fig. S6 Effects of CF101 or CF102 on the A_3AR mutants using NanoBiT association assay.

These residues in A_3AR formed hydrophobic interactions with the 3-iodobenzyl group present in CF101 and CF102. Data shown are mean ± S.E.M. of three independent experiments (n = 3).

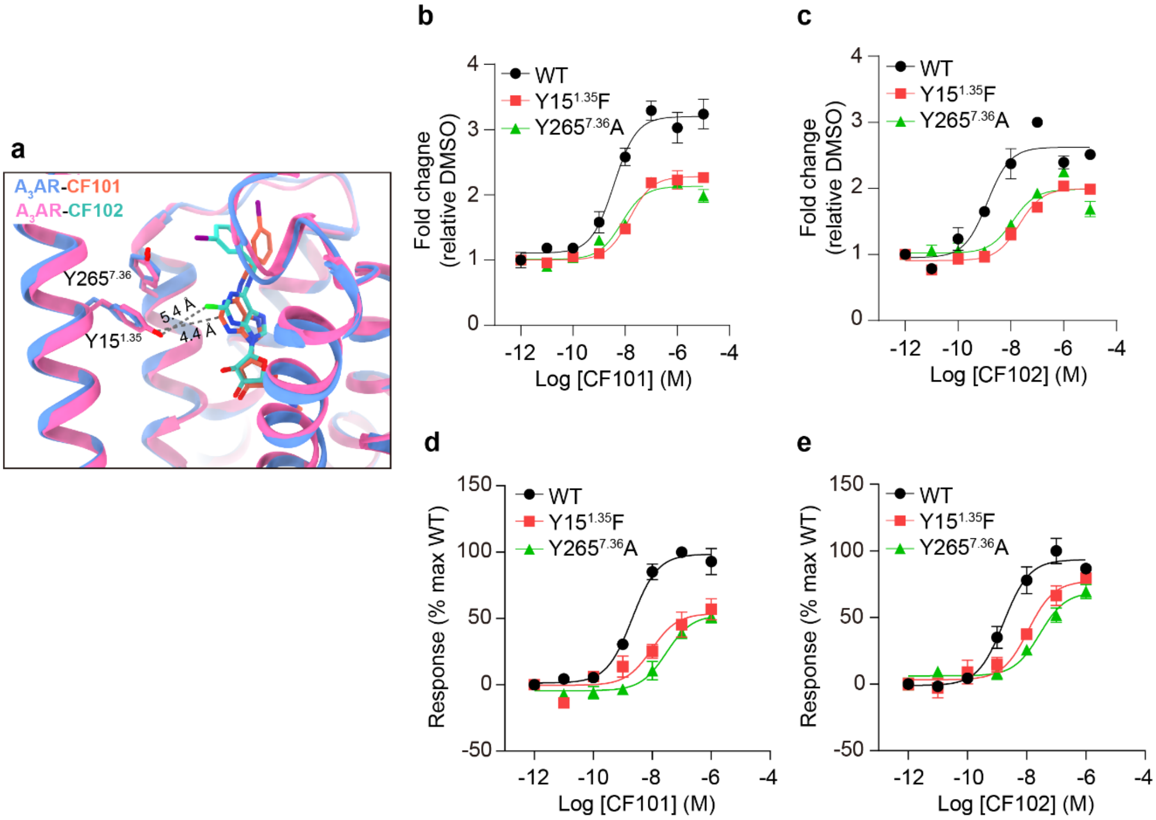

Fig. S7 The role of Y15$^{1.35}$ and Y265$^{7.36}$.

a Y15$^{1.35}$ formed hydrophobic interaction with Y265$^{7.36}$ in CF102-bound A_3AR. **b-e** Effects of CF101 and CF102 on the A_3AR and mutants of Y15$^{1.35}$F and Y265$^{7.36}$A using NanoBiT association assay (**b** and **c**) and cAMP accumulation assay (**d** and **e**), respectively. Data shown are mean ± S.E.M. of three independent experiments (n = 3). Source data are provided as a Source Data file.

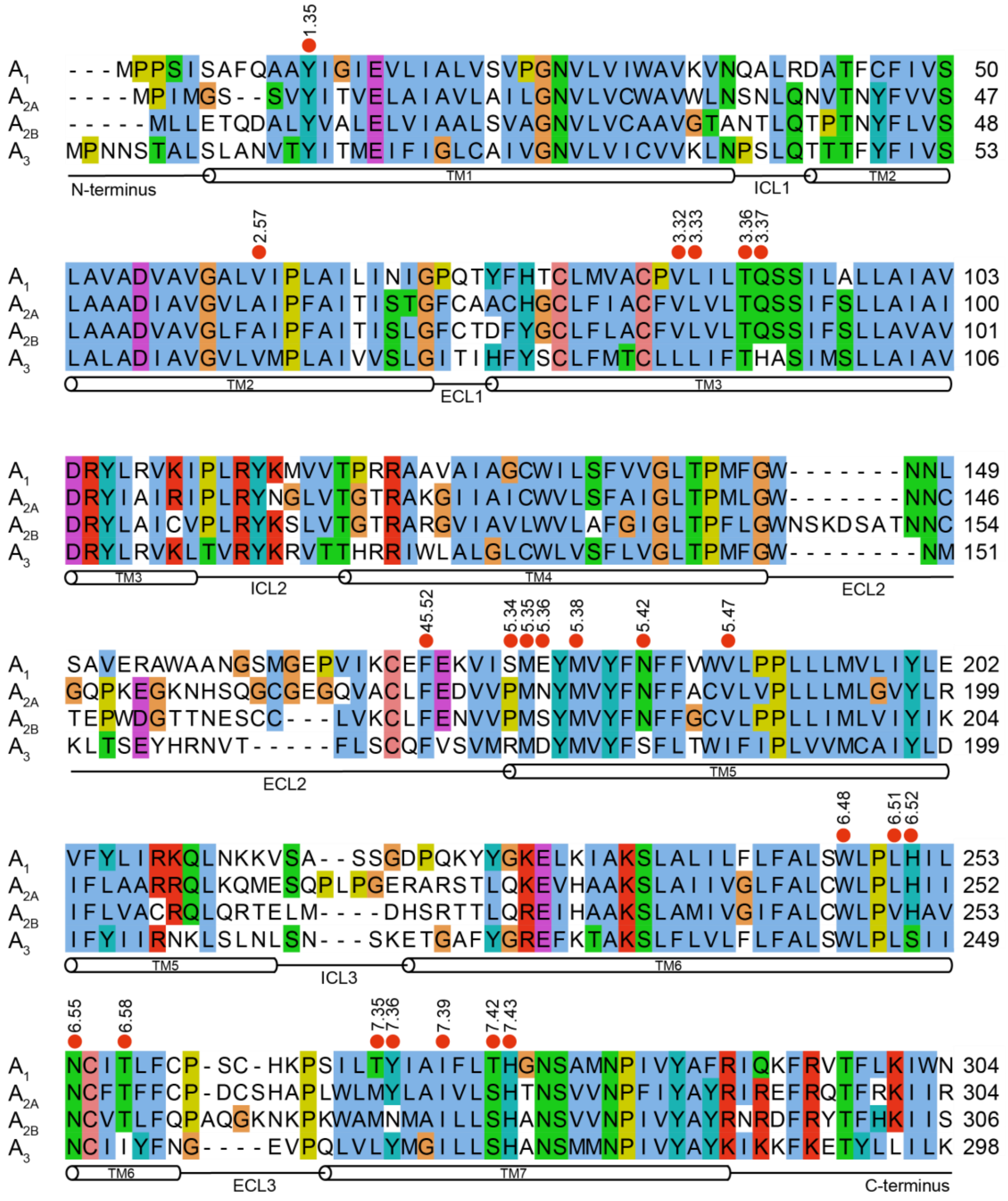

Fig. S8 Sequence alignment of adenosine receptors.

The sequence alignment was generated with Jalview[1] and depicts the N-/C-terminus, transmembrane helices (TMs), extracellular loops (ECLs), and intracellular loops (ICLs). Residues lining the orthosteric binding site are highlighted with red circles and annotated with GPCR Ballesteros-Weinstein numbering scheme. The C-terminus of the adenosine receptors were omitted.

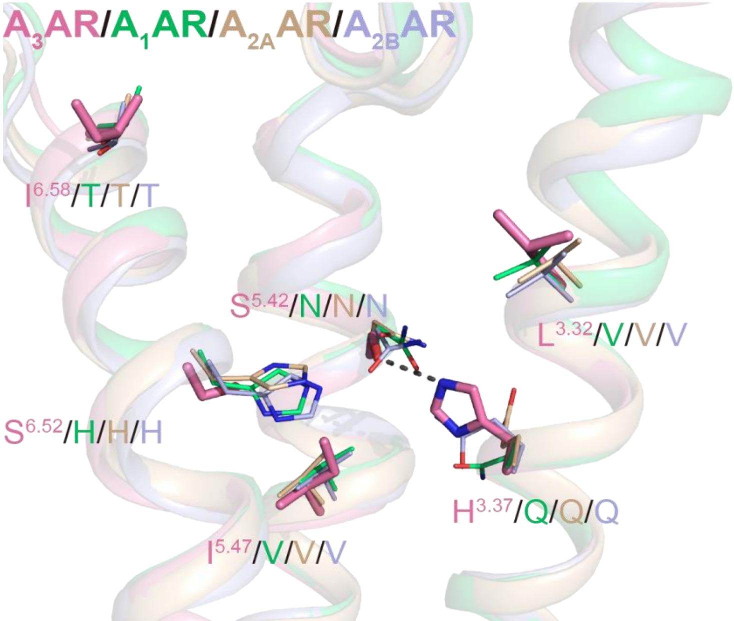

Fig. S9 The orthosteric binding pockets among adenosine receptors.

The positions highlighted indicate where unique residues occurred in A_3AR compared to other adenosine receptors. The receptor names and their associated colors were shown above the models. The side chains in A_3AR were depicted as bold sticks, while the corresponding side chains in other adenosine receptors were shown as thick sticks.

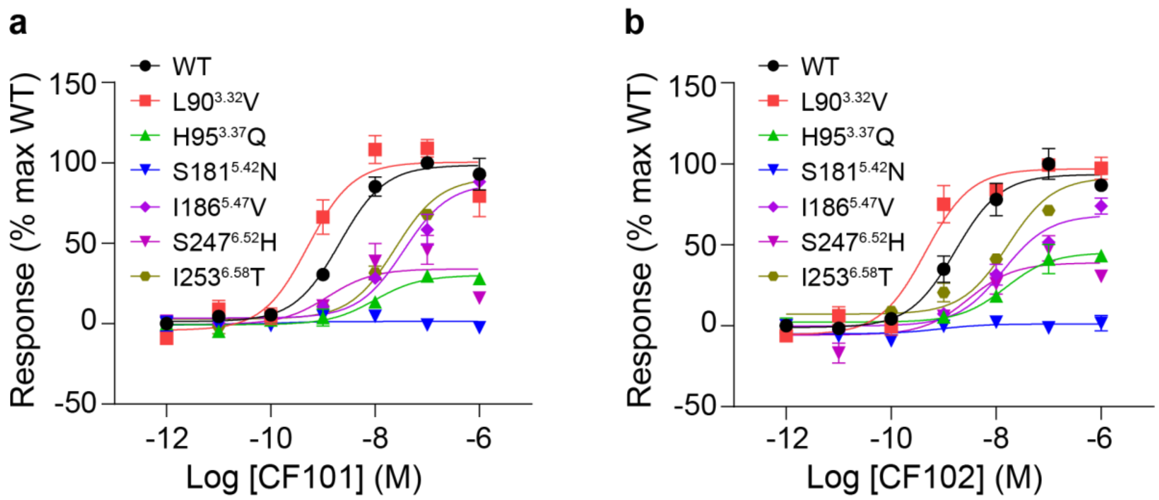

Fig. S10 Effects of CF101/CF102 on A$_3$AR mutants containing swapped residues from other adenosine receptors by cAMP accumulation assay. Data shown are mean ± S.E.M. of three independent experiments (n = 3). Source data are provided as a Source Data file.

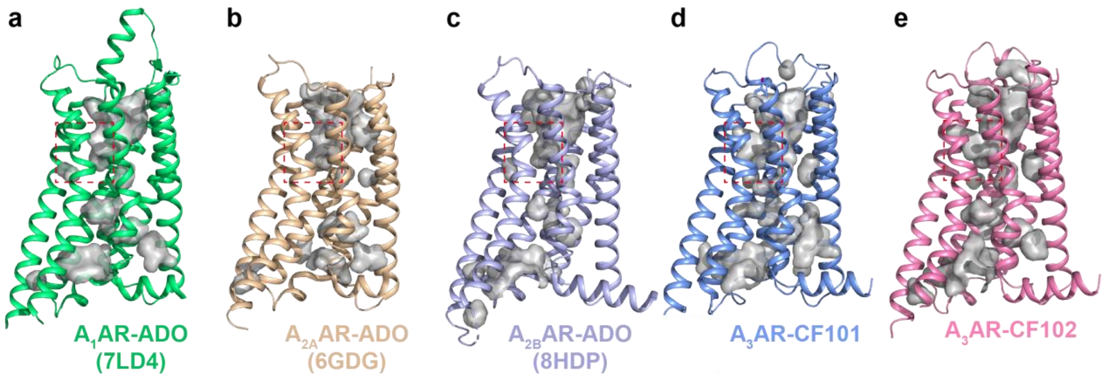

Fig. S11 The binding cavities in adenosine receptors.

a-e The binding cavities of the adenosine receptors are depicted as gray surfaces, with the bound ligands shown as sticks. The receptor names and associated PDB codes[2-4] are indicated below each model. The unique subpocket in the A3AR is indicated by the red boxes.

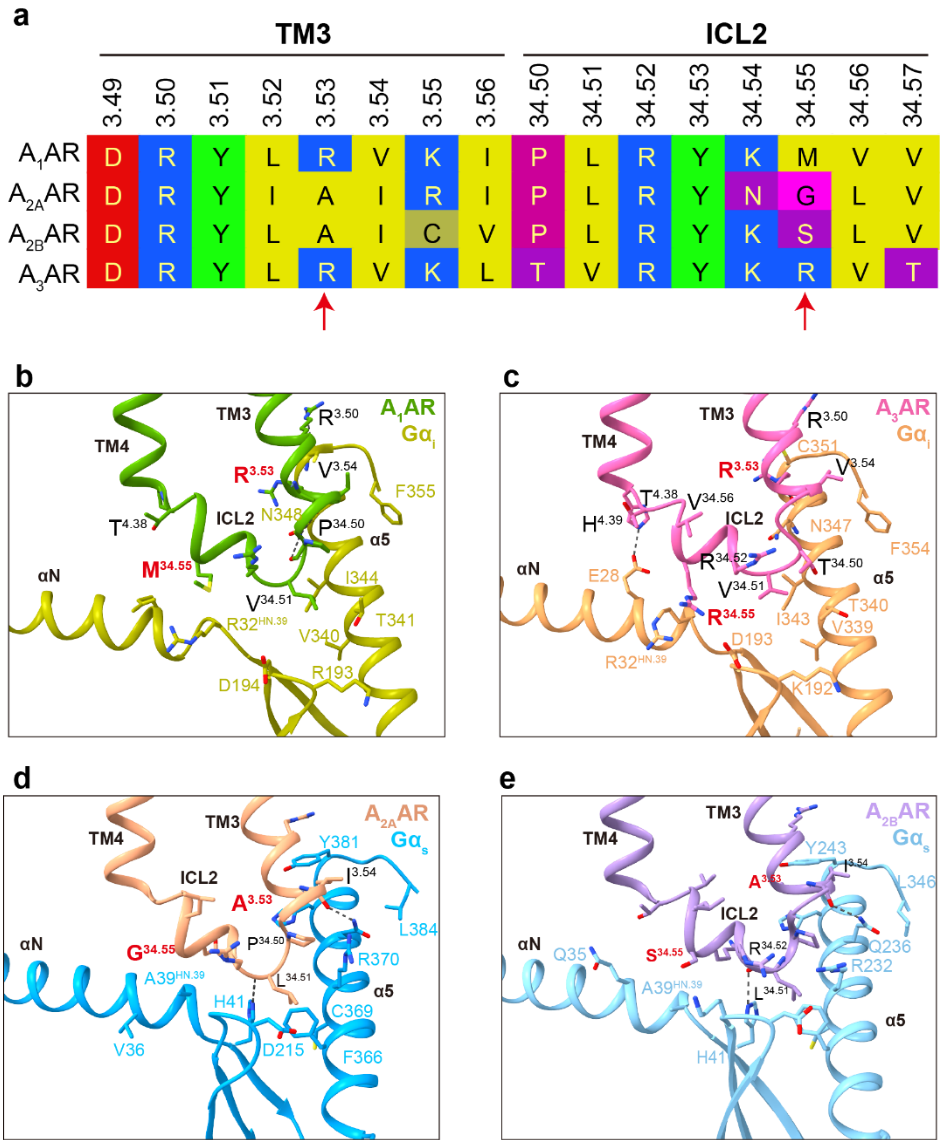

Fig. S12 Adenosine receptor-Gα protein interaction.

a sequence alignment of part of TM3 and ICL2 of adenosine receptors. The panel was generated on GPCRdb[5]. The red arrows indicate the unconserved residues. **b** A_1AR-Gα_i protein interaction. **c** A_3AR-Gα_i protein interaction. **d** A_{2A}AR-Gα_s protein interaction. **e** A_{2B}AR-Gα_s protein interaction. The polar interactions are indicated by black dashed lines.

Table S1 Cryo-EM data collection, model refinement and validation statistics.

	A_3AR-CF101-G_i complex	A_3AR-CF102-G_i complex
**Data collection and processing**		
Detector	K3	K3
Magnification	105,000	105,000
Voltage (kV)	300	300
Electron exposure ($e^–/Å^2$)	50	50
Defocus range (μm)	-1.0~-3.0	-1.0~-3.0
Pixel size (Å)	0.824	0.824
Symmetry imposed	C1	C1
Initial particle projections (no.)	9,970,197	8,924,341
Final particle projections (no.)	271,323	102,581
Map resolution (Å)	3.29	3.19
Map resolution range (Å)	2.60-4.40	2.40-4.20
FSC threshold	0.143	0.143
**Model Refinement**		
Refinement package	PHENIX-1.17.1-3660	PHENIX-1.17.1-3660
Real or reciprocal space	Real space	Real space
Model-Map CC (mask)	0.60	0.72
Model resolution (Å)	4.10	3.40
FSC threshold	0.5	0.5
B factors ($Å^2$, min/max/mean value)		
Protein residues	30.00/127.38/68.24	30.00/135.93/68.27
Ligands	20.00/20.00/20.00	20.00/20.00/20.00
**Model composition**		
Non-hydrogen atoms	8,751	8,753
Protein residues	1,126	1,127
R.m.s. deviations		
Bond lengths (Å)	0.001	0.005
Bond angles (°)	0.398	0.900
**Validation**		
MolProbity score	1.46	1.15
Clashscore	8.51	3.62
Rotamer outliers (%)	0.00	0.00
Ramachandran plot		
Favored (%)	98.19	98.38
Allowed (%)	1.81	1.62
Disallowed (%)	0.00	0.00
**Data availability**		
EMDB entry	8X16	8X17
PDB entry	EMD-37985	EMD-37986

Table S2 Cell surface expression of A_3AR and its mutants on CF101- and CF102-induced NanoBiT assay.

	pEC$_{50}$[a]				Cell-surface expression (Relative to WT)[a]	
	CF101		CF102			
		P value		*P* value		*P* value
WT	8.44 ± 0.09		8.86 ± 0.13		100.0 ± 2.5	
Y15$^{1.35}$A	UD[b]		UD		112.1 ± 5.0	0.6748
Y15$^{1.35}$F	7.83 ± 0.05	0.0283	7.71 ± 0.09***	<0.0001	172 ± 3.5***	<0.0001
L90$^{3.32}$V	8.58 ± 0.08	0.9699	8.41 ± 0.06	0.1044	79.6 ± 6.8	0.1092
L91$^{3.33}$A	UD		UD		106.9 ± 12.1	0.9943
T94$^{3.36}$A	UD		UD		91.5 ± 4.7	0.9824
H95$^{3.37}$A	UD		UD		121.2 ± 7.1	0.0858
H95$^{3.37}$Q	8.30 ± 0.33	0.9669	8.36 ± 0.17	0.059	123.7 ± 5.6	0.0359
F168^{ECL2}A	<5		UD		116.2 ± 4.0	0.3482
V169^{ECL2}A	NT[c]		9.00 ± 0.12	0.9525	85.9 ± 6.0	0.5408
M174$^{5.35}$A	8.73 ± 0.10	0.5586	NT		85.0 ± 2.3	0.4522
M177$^{5.38}$A	<5		UD		110.5 ± 1.4	0.8877
S181$^{5.42}$A	UD		UD		102.2 ± 5.3	0.9996
S181$^{5.42}$N	UD		UD		88.8 ± 4.1	0.8298
I186$^{5.47}$A	UD		UD		79.2 ± 2.7	0.0979
I186$^{5.47}$V	7.99 ± 0.10	0.1478	7.55 ± 0.12***	<0.0001	93.6 ± 2.3	0.9951
W243$^{6.48}$A	<5		UD		65.5 ± 6.2**	0.0003
L246$^{6.51}$A	<5		UD		81.0 ± 4.9	0.1661
S247$^{6.52}$H	<5		UD		82.5 ± 6.2	0.2547
N250$^{6.55}$A	UD		UD		78.2 ± 5.0	0.07
I253$^{6.58}$T	7.79 ± 0.10	0.0179	7.95 ± 0.19**	0.0005	90.1 ± 9.8	0.9268
L264$^{7.35}$A	8.41 ± 0.09	0.5015	8.29 ± 0.12**	0.0004	73.7 ± 3.8	0.0127
Y265$^{7.35}$A	8.14 ± 0.04	0.9997	7.94 ± 0.05	0.0255	90.4 ± 1.2	0.9429
I268$^{7.39}$A	UD		UD		67.4 ± 4.2**	0.0008
H272$^{7.43}$A	UD		UD		71.4 ± 2.3*	0.0048

[a] Data shown are means ± S.E.M. from at least three independent experiments.

[b] UD indicates that the activation level is too low to determine pEC$_{50}$ values.

[c] NT, not test.

* *P*<0.01; ***P*<0.001 and ****P*<0.0001 by one-way ANOVA followed by multiple comparisons test, compared with WT.

Table S3 Cell surface expression of A_3AR and its mutants on CF101- and CF102-induced cAMP assay.

	pEC50[a]				Cell-surface expression (Relative to WT)[a]	
	CF101		CF102			
		P value		*P* value		*P* value
WT	8.66 ± 0.07		8.73 ± 0.19		100 ± 1.1	
Y15$^{1.35}$A	UD		UD		78.5 ± 1.5***	<0.0001
Y15$^{1.35}$F	8.13 ± 0.42	0.5945	7.96 ± 0.05	0.1159	111.8 ± 2.2***	<0.0001
L90$^{3.32}$V	9.29 ± 0.12	0.4368	9.33 ± 0.14	0.3344	93.8 ± 1.0	0.0267
L91$^{3.33}$A	UD[b]		7.48±0.32*	0.0039	108 ± 1.5**	0.0006
T94$^{3.36}$A	UD		UD		93.7±1.1	0.0226
H95$^{3.37}$A	UD		UD		117±1.2***	<0.0001
H95$^{3.37}$Q	8.29 ± 0.46	0.8656	7.72 ± 0.17	0.0239	114 ± 0.92***	<0.0001
F168^{ECL2}A	<6		7.09 ± 0.41**	0.0002	120 ± 1.1***	<0.0001
M177$^{5.38}$A	<6		6.45 ± 0.21***	<0.0001	104 ± 1.2	0.2355
S181$^{5.42}$A	UD		UD		93.2 ± 0.89	0.0104
S181$^{5.42}$N	UD		UD		110 ± 0.93**	<0.0001
I186$^{5.47}$A	UD		UD		102 ± 1.6	0.9803
I186$^{5.47}$V	7.34 ± 0.30	0.0166	7.88 ± 0.20	0.0694	101 ± 0.86	0.9991
W243$^{6.48}$A	<6		<6		82.0 ± 0.73***	<0.0001
L246$^{6.51}$A	UD		UD		89.7 ± 0.52***	<0.0001
S247$^{6.52}$H	8.61 ± 0.18	0.9998	8.39 ± 0.19	0.8574	101 ± 1.5	0.9993
N250$^{6.55}$A	UD		UD		106 ± 1.4	0.0424
I253$^{6.58}$T	7.61 ± 0.10	0.0656	7.82 ± 0.12	0.0484	93.1 ± 3.1*	0.0091
Y265$^{7.36}$A	7.60 ± 0.17	0.0633	7.53 ± 0.14	0.0055	103 ± 1.0	0.847
I268$^{7.39}$A	UD		UD		89.6 ± 0.77***	<0.0001
H272$^{7.43}$A	UD		UD		39.7 ± 1.3***	<0.0001

[a] Data shown are means ± S.E.M. from at least three independent experiments.

[b] UD indicates that the activation level is too low to determine pEC_{50} values.

* *P*<0.01; ***P*<0.001 and ****P*<0.0001 by one-way ANOVA followed by multiple comparisons test, compared with WT.

Table S4 Cell surface expression of $A_1AR/A_{2A}AR/A_{2B}AR$ and its relative mutant on CF101- and CF102-induced NanoBiT assay.

		A_1AR	A_1AR-ECL3	$A_{2A}AR$	$A_{2A}AR$-ECL3	$A_{2B}AR$	$A_{2B}AR$-ECL3
Adenosine	pEC_{50}[a]	5.27±0.16	5.26±0.15	4.9±0.21	4.68±0.02	4.57±0.09	4.91±0.21
	P value		>0.9999		0.7786		0.4382
	Emax[a]	3.98±0.69	4.08±0.53	1.96±0.03	2.25±0.07	3.06±0.12	2.78±0.21
	P value		0.9997		0.9725		0.9723
CF101	pEC_{50}[a]	6.71±0.11	6.82±0.11	6.05±0.26	7.15±0.09*	<5	5.72±0.06
	P value		0.9612		0.0011		
	Emax[a]	1.19±0.1	3.69±0.2***	1.53±0.1	2.21±0.1	1.77±0.1	4.06±0.2***
	P value		<0.0001		0.0385		<0.0001
CF102	pEC_{50}[a]	<5	6.95±0.30	UD[b]	6.97±0.11	<5	<5
	P value						
	Emax[a]	2.12±0.1	3.86±0.6*	1.03±0.1	1.84±0.1	0.95±0.1	2.72±0.2*
	P value		0.0018		0.1591		0.0016
	Expression[c]	100±1.5	91.1±3.2	100±2.9	92.1±1.6	100±7.6	80.5±1.9*
	P value		0.3346		0.4426		0.004

[a] Data shown are means ± S.E.M. from at least three independent experiments.

[b] UD indicates that the activation level is too low to determine pEC_{50} values.

[c] The expression indicates the cell-surface expression which was relative to the wild type.

* *P*<0.01; ***P*<0.001 and ****P*<0.0001 by one-way ANOVA followed by multiple comparisons test, compared with WT.

References

1. Waterhouse, AM, JB Procter, DM Martin, M Clamp and GJ Barton, Jalview Version 2--a multiple sequence alignment editor and analysis workbench. *Bioinformatics*, 2009. 25(9): 1189-91.
2. Garcia-Nafria, J, Y Lee, X Bai, B Carpenter and CG Tate, Cryo-EM structure of the adenosine A(2A) receptor coupled to an engineered heterotrimeric G protein. *Elife*, 2018. 7.
3. Draper-Joyce, CJ, R Bhola, J Wang, A Bhattarai, ATN Nguyen, I Cowie-Kent, K O'Sullivan, LY Chia, et al., Positive allosteric mechanisms of adenosine A(1) receptor-mediated analgesia. *Nature*, 2021. 597(7877): 571-576.
4. Cai, H, Y Xu, S Guo, X He, J Sun, X Li, C Li, W Yin, et al., Structures of adenosine receptor A(2B)R bound to endogenous and synthetic agonists. *Cell Discov*, 2022. 8(1): 140.
5. Horn, F, E Bettler, L Oliveira, F Campagne, FE Cohen and G Vriend, GPCRDB information system for G protein-coupled receptors. *Nucleic Acids Res*, 2003. 31(1): 294-7.
